# FGF21 Is Released During Increased Lipogenesis State Following Rapid-Onset Radioiodine-Induced Hypothyroidism

**DOI:** 10.3389/fendo.2022.900034

**Published:** 2022-07-14

**Authors:** Ewa Szczepańska, Piotr Glinicki, Wojciech Zgliczyński, Jadwiga Słowińska-Srzednicka, Helena Jastrzębska, Małgorzata Gietka-Czernel

**Affiliations:** Department of Endocrinology, Bielański Hospital, Centre of Postgraduate Medical Education, Warsaw, Poland

**Keywords:** FGF21, hyperthyroidism, hypothyroidism, SHBG, triglycerides, lipogenesis, NAFLD

## Abstract

**Background:**

FGF21 pharmacological treatment reverses fatty liver and lowers serum triglyceride concentration but FGF21 serum level is increased in hepatic steatosis. FGF21 secretion is induced by thyroid hormones *in vitro*.

**Purpose:**

To determine the influence of thyroid hormones and metabolic changes secondary to thyroid dysfunction on FGF21 secretion in humans.

**Materials and Methods:**

This was a case-control study. 82 hyperthyroid and 15 hypothyroid patients were recruited together with 25 healthy controls. Of those with hyperthyroidism, 56 received radioiodine treatment and 42 of them achieved hypothyroidism and then euthyroidism within one year following therapy. Radioiodine-induced hypothyroidism developed abruptly within a six week interval between clinic visits. FGF21 serum levels were determined with an ELISA method.

**Results:**

Serum FGF21 levels did not differ in hyper- and hypothyroid patients in comparison to controls [median 103.25 (interquartile range, 60.90-189.48) and 86.10 (54.05-251.02) vs 85.20 (58.00-116.80) pg/mL P=0.200 and 0.503, respectively]. In hyperthyroid patients treated with radioiodine, serum FGF21 levels increased significantly in rapid-onset hypothyroidism in comparison to the hyperthyroid and euthyroid phase [median 160.55 (interquartile range, 92.48 - 259.35) vs 119.55 (67.78-192.32) and 104.43 (55.93-231.93) pg/mL, P=0.034 and 0.033, respectively]. The rising serum FGF21 level correlated positively with serum triglycerides (Spearman coefficient rs=0.36, P=0.017) and inversely with serum SHBG (rs=-0.41, P=0.007), but did not correlate with thyroid hormone levels.

**Conclusions:**

There was a transient increase in FGF21 serum level during rapid-onset hypothyroidism following radioiodine treatment. There was no association between FGF21 serum level and thyroid hormones. In radioiodine-induced hypothyroidism, the rising serum FGF21 concentration correlated positively with rising serum triglycerides and negatively with falling SHBG, reflecting increased hepatic lipogenesis.

## Introduction

Thyroid hormones increase basal metabolic rate, stimulate brown adipose tissue thermogenesis, and reduce cholesterol and triglyceride levels. Body mass and lipid changes are essential symptoms of thyroid dysfunction, resulting directly from altered thyroid hormone levels, but also from cross-talk with numerous metabolic factors. Fibroblast growth factor 21 (FGF21) is a novel potent metabolic regulator which actions have considerable overlap with thyroid hormones. Similar to thyroid hormones, FGF21 lowers serum lipid levels, stimulates brown adipose tissue thermogenesis, and promotes weight loss (1–3). Its secretion is induced by T3 in murine and human hepatic cell culture (4, 5). However, the stimulating effect of thyroid hormones on FGF21 release in humans is controversial, with studies demonstrating divergent results (6, 7).

FGF21 together with FGF19, and FGF23 belong to the fibroblast growth factor family with hormone-like activity. In contrast to classical fibroblast growth factors that act in an auto- and paracrine manner, hormone-like FGFs are released into the bloodstream and exert endocrine action in distant tissues (8, 9). Serum FGF21 is generated primarily in the liver (10) under nutritional stress stimuli like starvation (11, 12) or a lipotoxic diet (12–15), but also increased mitochondrial and endoplasmic reticulum stress (16, 17). Specifically in the liver, it produces essential biological action of protecting hepatocytes from metabolic stress caused by fat overload (18). FGF21 stimulates hepatic fatty acid oxidation instead of its conversion into triglycerides (19). Moreover, FGF21 reduces the flow of lipids into the liver by increased peripheral lipoprotein catabolism (20) and reduced adipocytes lipolysis (21). Exogenous FGF21 administration reduces hepatic fat content and reverses fatty liver (2, 21–24). Paradoxically, although some studies suggest direct paracrine action of FGF21 in the liver (25), others demonstrate the lack of FGF21 key receptor FGFR1c in hepatocytes, suggesting that the decrease in hepatic triglyceride deposition may be mediated indirectly (26). It has been proposed that the release of adiponectin from adipose tissue in response to liver-derived FGF21 reduces hepatic lipid content in a feedback manner (27). However, other research has demonstrated adiponectin is dispensable for FGF21 action (28), or even conversely adipokine generated in adipocytes induces FGF21 expression in the liver (29). However, this data has emerged from *in vitro* and animal research, or clinical studies following administration of FGF21 analogues, and so does not fully reflect physiology.

Besides plasma and hepatic lipid regulation, FGF21 exerts the critical action of controlling energy homeostasis (1, 2, 28, 30). Serum FGF21 derives predominantly, if not only from the liver (10) and exerts its action in the central nervous system (31, 32) and adipose tissue (28, 33, 34). Acting in the ventromedial hypothalamus, FGF21 diminishes sweet-taste preference that results in simple sugar intake suppression (35). In adipose tissue, FGF21 promotes glucose utilization and increases energy expenditure by enhancing insulin sensitivity, stimulating browning of white adipocytes and brown adipose tissue thermogenesis (28, 33, 34). Therefore, FGF21 induces weight loss by suppression of sucrose intake, and increase in brown adipose tissue insulin sensitivity resulting in glucose consumption for heat production instead of energy storage. Paradoxically despite its beneficial action, FGF21 is elevated in insulin resistance states i.e. fatty liver, obesity, and type 2 diabetes (36–38). It is not clear if this effect results from FGF21 resistance or compensatory increased secretion (38, 39).

The aim of the study was to investigate whether thyroid hormones directly change FGF21 secretion in hyper- and hypothyroidism, or whether metabolic challenge related to considerable changes in glucose and lipid metabolism and body composition that accompany thyroid dysfunction indirectly affect FGF21 release. Therefore, we firstly analyzed FGF21 serum levels in hyper-and hypothyroid subjects as compared to the euthyroid control group. Secondly, we analyzed circulating FGF21 in patients with Graves’ disease that were treated with radioiodine (RAI), in three different thyroid function states, namely hyperthyroidism at clinic referral, hypothyroidism induced by RAI treatment that developed abruptly within a six week interval between clinic visits, and euthyroidism after l-thyroxine treatment. These typically occur in 80% of patients within one year of undergoing treatment. Additionally, we analyzed serum adiponectin levels and its association with circulating FGF21 in the different thyroid function states. These patients were chosen because of dramatic thyroid hormone, lipid, and body composition changes that occurred in a short period of time, which triggers different metabolic counterregulatory pathways e.g. possibly FGF21 secretion. Finally, we analyzed a subgroup of thionamide-treated hyperthyroid patients pre-treatment, and three months after euthyroidism had been established.

## Materials and Methods

### Study Participants

We performed a case-control study enrolling 82 hyperthyroid patients with Graves’ disease and 15 hypothyroid patients with Hashimoto’s thyroiditis from the Department of Endocrinology, Centre of Postgraduate Medical Education, Bielański Hospital (Warsaw, Poland), from September 2016 to September 2020. The control group consisted of 25 healthy hospital employees. The study protocol was approved by the Bioethical Committee of Centre of Postgraduate Medical Education, Warsaw, Poland. All participants provided written informed consent.

The inclusion criteria were as follows: (1) age ≥ 18 years (2) body mass index (BMI) ≤ 35 (3) hyperthyroidism in the course of Graves’ disease (4) hypothyroidism in the course of Hashimoto’s thyroiditis. The control group was recruited from healthy volunteers that were free of thyroid disease. The exclusion criteria were as follows: (1) diabetes (2) cancer (3) pregnancy and lactation (4) abnormal hepatic function tests (elevated alanine aminotransferase (ALT) or aspartate aminotransferase (AST) >1.5 times above reference value) (5) renal dysfunction (defined glomerular filtration rate [GFR] <60 ml/min/1.73 kg\m^2^) (6) acute cardiorespiratory or infectious disease.

The diagnosis of hyperthyroidism in the course of Graves’ disease was established based on the typical clinical characteristics, including decreased thyroid stimulating hormone (TSH), elevated thyroid hormones (TH) and TSH receptor antibody (TRAb) serum levels. Hypothyroidism in the course of Hashimoto’s thyroiditis was diagnosed based on clinical presentation, elevated serum TSH, decreased TH levels, and the presence of thyroid peroxidase antibody (TPOAb).

### Study Design

In all patients with hyper- and hypothyroidism and the control group, measurements were performed to determine: FGF21, adiponectin, TSH, TH, sex hormone-binding globulin (SHBG), lipids, glucose, insulin, and body composition at baseline.

**Figure 1:**
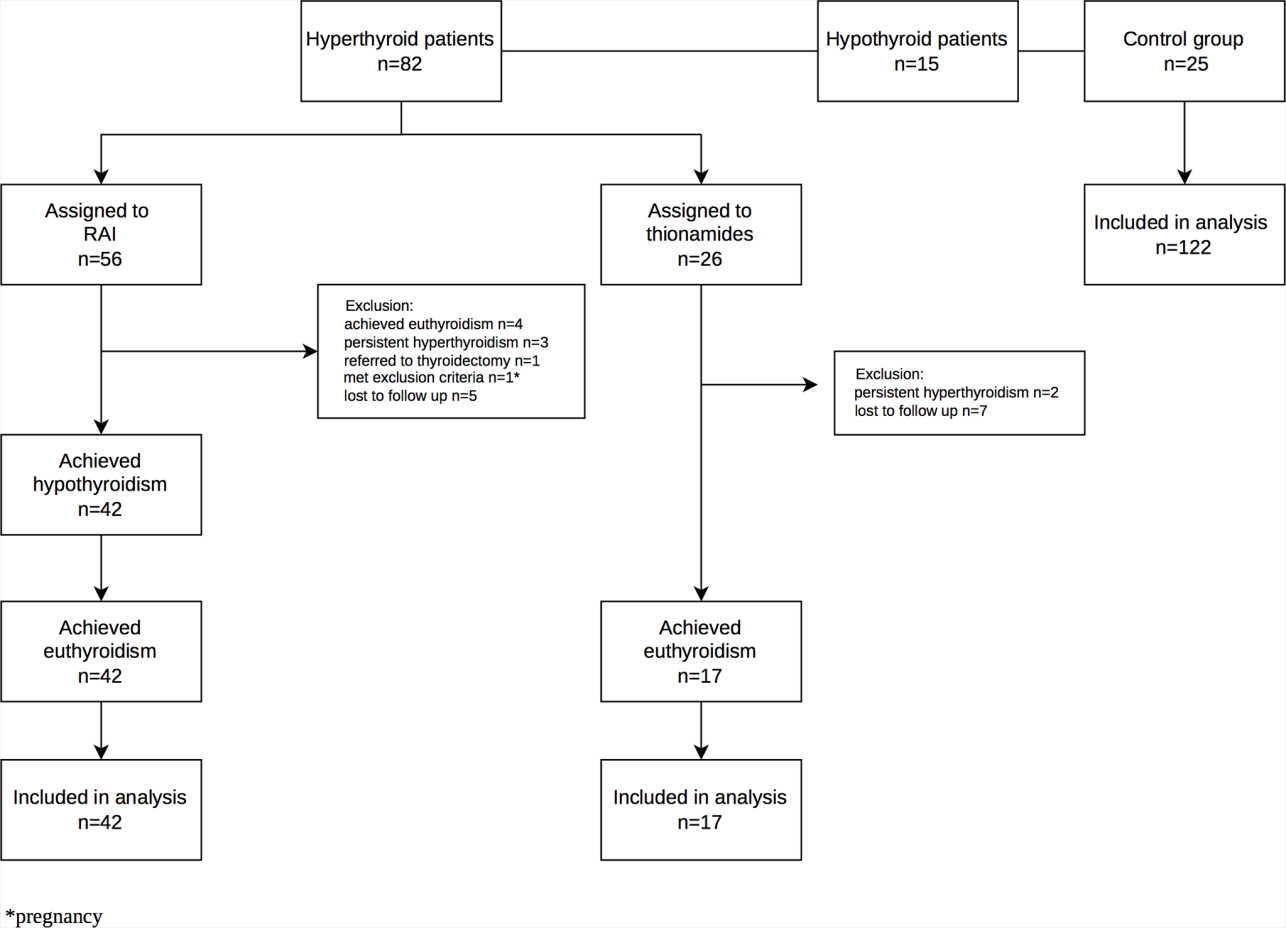
Flow diagram of the participant in the case-control study.

Of the hyperthyroid patients, 56 were assigned to RAI treatment according to clinical indications and patient choice, whereas the remaining 26 were treated with thionamides. The patients treated with RAI were observed every six weeks until hypothyroidism and finally euthyroidism occurred following l-thyroxine treatment. In these patients, the above-mentioned biochemical and body composition measurements were performed at three time-points, namely initially during the hyperthyroid phase, secondly at the hypothyroid phase, and finally upon reaching euthyroidism while treated with l-thyroxine after three months of successful replacement therapy. Additionally, in the thionamide treated group, the measurements were performed pre-treatment and three months after euthyroidism had been established (**Figure 1**).

### Anthropometric Measures and Laboratory Analyses

Body weight was measured in the morning using an electronic platform scale (Marsden BFA-220P, UK). Height was measured using a wall-mounted stadiometer. Body mass index (BMI) was calculated as body weight (kilograms) divided by height (meters^2^). Waist circumference was measured in the midpoint between the lowest rib and the superior margin of the iliac crest.

All patients with hyper- and hypothyroidism, and healthy controls had blood drawn from the antecubital vein after 12h of fasting, 8.00-9.00 AM. The blood was centrifuged, and serum frozen at -80°C for FGF21 and adiponectin analysis.

Serum TSH was determined using chemiluminescence immunoassay (UniCel DxI 600, Beckman Coulter, USA). Serum fT4, fT3, insulin, SHBG, TPOAb, thyroglobulin antibodies (TGAb) were determined using a chemiluminescence immunoassay (Liaison^®^, DiaSorin, Italy). Serum TRAb was determined with electrochemiluminescence assay (Cobas 8000, Roche Diagnostics, Switzerland).

Serum AST, ALT, creatinine, cholesterol, HDL-cholesterol (HDL-c), and triglycerides (TG) were determined on a biochemical analyzer (Cobas 6000, Roche Diagnostics, Switzerland). Plasma glucose was measured using the hexokinase method (Cobas 6000, Roche Diagnostics, Switzerland).

LDL-cholesterol (LDL-c) was calculated using the Friedewald equation. Insulin sensitivity was assessed using the homeostasis model of assessment for insulin resistance (HOMA-IR) and quantitative insulin sensitivity check index (Quicki). HOMA-IR was calculated according to the formula: fasting insulin (µIU/ml) × fasting plasma glucose (FPG, mmol\l)/22.5 and Quicki was calculated as follows: 1/(log (fasting insulin µIU/ml) + log (fasting glucose mg/dl)).

Intact serum FGF21 concentration was determined using the ELISA kit (Epitope Diagnostics, USA, Cat# KT-879, RRID: AB_2895552) according to the manufacturer’s instructions. The detectable range of the assay was 1.7-2169 pg/ml. The intra- and inter-assay coefficients of variation reported by the manufacturer were 5.7%- 6.9% respectively. This assay is highly specific for human intact FGF21 and do not cross react with FGF21 fragments.

Serum adiponectin concentration was assessed using the ELISA kit (Mediagnost, Germany, Cat# E09, RRID : AB_2813736). The detectable range of the assay was 0.27-31000 g/l and the intra- and inter-assay coefficients of variation reported by the manufacturer were 5%-7.5%, respectively. This assay is highly specific for human total adiponectin.

### Body Composition Measurement

Body composition was assessed with a whole-body fan-beam dual energy X-ray absorptiometry (DXA) scan (Lunar Prodigy Advance, software v 11.40, GE Healthcare, USA). Body fat (BF), lean tissue mass (LTM), and bone mineral content (BMC) were analyzed using system software. Body regions (arms, legs, trunk, and head) were delineated and BF of particular regions and the total body were analyzed using system software.

### Statistical Analysis

All statistical analyses were performed using the R statistic package, version 4.0.5. (http://cran.r-project.org). Shapiro-Wilk’s test was used to assess the normality of data distribution, and log-transformation was applied to non-normally distributed variables and normality retested. Levene’s test was used to assess the equality of variances across groups, the chi-squared test was used to assess the equality of group sizes. Mauchly’s sphericity test was used to assess the sphericity of variances. All measurements are presented as means ± SD for normally distributed data or median (interquartile range) for non-normally distributed data.

Fisher’s exact test was used to compare categorical variables between groups. Student’s unpaired t-test was used to compare normally distributed continuous variables and the Mann-Whitney U-test was used to compare non-normally distributed continuous variables. Changes in FGF21 serum levels and other measures in pair-wise comparison after RAI treatment were examined using a repeated measures ANOVA for normally distributed data and Wilcoxon-signed-rank test for non-normally distributed data even after being logarithmically transformed. For statistically significant ANOVA analyses, *post-hoc* Student’s paired t-tests were used to find individual points of significance. A Bonferroni correction for multiple comparisons was applied. We used a paired Student’s t-test for normally distributed data or Wilcoxon-signed-rank test for non-normally distributed data to compare variables in thionamide treated group. Relationships between continuous variables were estimated by Spearman correlation coefficient analysis, because the majority of the datasets were not normally distributed. *P* < 0.05 were considered significant.

## Results

### Baseline Characteristics of the Subjects

Baseline characteristics of the subjects are shown in **Table 1**. One person from the control group and one patient with hyperthyroidism were excluded because of the extremely high baseline serum FGF21 values exceeding the upper range of assay sensitivity. Studied groups and control were matched for age, gender and BMI distribution. TSH and TH levels were different between groups according to different thyroid functions. SHBG serum level was higher in hyperthyroid patients compared to the control group. Plasma glucose and serum insulin concentration, HOMA-IR, and Quicki were comparable between patients and controls. As expected, serum total cholesterol, and LDL-c levels were lower in patients with hyperthyroidism, and higher in hypothyroidism, whereas HDL-c was lower in hyperthyroidism. Unlike cholesterol, serum TG levels in hyperthyroid and hypothyroid patients did not differ compared to controls. Additionally, AST was higher in hyperthyroidism and GFR lower in hypothyroidism and higher in hyperthyroidism compared to the control group (**Table 1**). There was significantly lower LTM in hyperthyroid women than in healthy controls, whereas other body composition parameters in both sexes were comparable (**Suppl. Tables 1**, **2**).

**Table 1:** Serum FGF21 and biochemical parameters in the study groups and controls.

	Controls	Hyperthyroidism	*P^α^*	Hypothyroidism	*P^β^*
*N*	25	82		15	
Age, years	39.60 ± 10.62	44.50 ± 14.90	0.074	44.67 ± 14.14	0.242
*Gender, female, N(%)*	21 (84.0)	72 (87.8)	0.877	11 (73.3)	0.683
TSH, mIU/L	1.39 (1.06-1.89)	0.00 (0.00-0.00)	<0.001	97.00 (68.68-150.0)	<0.001
fT4, pmol/L	13.40 ± 1.82	56.57 ± 29.20	<0.001	6.94 ± 2.94	<0.001
fT3, pmol/L	4.51 ± 0.44	20.49 ± 10.07	<0.001	2.84 ± 0.98	<0.001
SHBG, nmol/L	62.03 ± 35.89	165.07 ± 64.68	<0.001	47.85 ± 22.33	0.137
Fasting serum insulin, mU/L	6.63 ± 3.55	7.13 ± 4.57	0.577	6.15 ± 3.36	0.677
Fasting plasma glucose, nmol/L	5.08 ± 0,56	5.33 ± 0,55	0.053	5.06 ± 0.59	0.924
Quicki	0.38 ± 0.03	0.37 ± 0.03	0.757	0.38 ± 0.05	0.338
HOMA-IR	1.11 (0.93-1.89)	1.31 (1.02-1.93)	0.455	1.20 (0.90-1.86)	0.817
TC, mmol/L	4.82 ± 0,56	3.81 ± 0,95	<0.001	6.35 ± 2.13	0.020
HDL-c, mmol/L	1.66 ± 0.35	1.39 ± 0.43	0.003	1.69 ± 0.46	0.796
LDL-c, mmol/L	2.69 ± 0.55	1.89 ± 0.71	<0.001	4.12 ± 1.75	0.010
TG, mmol/L	1.04 ± 0,4	1.13 ± 0.46	0.318	1.16 ± 0.37	0.317
BMI, kg/m^2^	24.20 ± 4.26	23.25 ± 3.69	0.316	25.10 ± 5.17	0.574
TPOAb, U/mL	0.75 (0.40-1.35)	329.20 (32.50-901.4)	<0.001	739.80 (72.20-1228.5)	<0.001
TGAb, U/ml	0.00 (0.00-0.43)	4.95 (0.68-46.45)	<0.001	322.00 (51.90-500.00)	<0.001
TRAb, U/l	<0.8	18.52 ± 12.68	<0.001	12.45 ± 16.48	<0.001
AST, U/L	18.36 ± 5.19	21.84 ± 6.92	0.029	23.94 ± 10.89	0.086
ALT, U/L	19.92 ± 10.77	24.32 ± 10.54	0.139	25.22 ± 12.08	0.214
Creatinine, µmol/L	65.43 ± 15.03	52.17 ± 15.03	0.003	76.93 ± 16.8	0.071
GFR, ml/min/1,73m^2^	90.00 (84.79-90.00)	90.00 (90.00-90.00)	0.020	79.23 (68.49-86.50)	0.011
Adiponectin, µg/mL	9.75 ± 5.57	11.67 ± 7.26	0.171	13.09 ± 8.34	0.195
FGF21,pg/mL	85.20 (58.00-116.80)	103.25 (60.90-189.48)	0.200	86.10 (54.05-251.02)	0.503

TSH, thyroid stimulating hormone; fT4, free thyroxine; fT3, free triiodothyronine; SHBG, sex hormone binding globulin; Quicki, quantitative insulin-sensitivity check index; HOMA-IR, homeostasis model assessment of insulin resistance; TC, total cholesterol; HDL-c, high density lipoprotein cholesterol; LDL-c, low density lipoprotein cholesterol; TG, triglycerides; BMI, body mass index; TPOAb, thyroid peroxidase antibody; TGAb, thyroglobulin antibody;

TRAb, TSH receptor antibody; AST, aspartate aminotransferase; ALT, alanine aminotransferase: GFR, glomerular filtration rate; FGF21, fibroblast growth factor 21. Data shown are N (%) for categorical variables and mean ± SD or median (interquartile range) for continuous variables.

Pα-values are based on comparison of characteristics between hyperthyroid patients vs controls.

Pβ-values are based on comparison of characteristics between hypothyroid patients vs controls.

### Serum FGF21 Levels in Hyper- and Hypothyroidism did not Differ in Comparison to the Control Group

**Figure 2:**
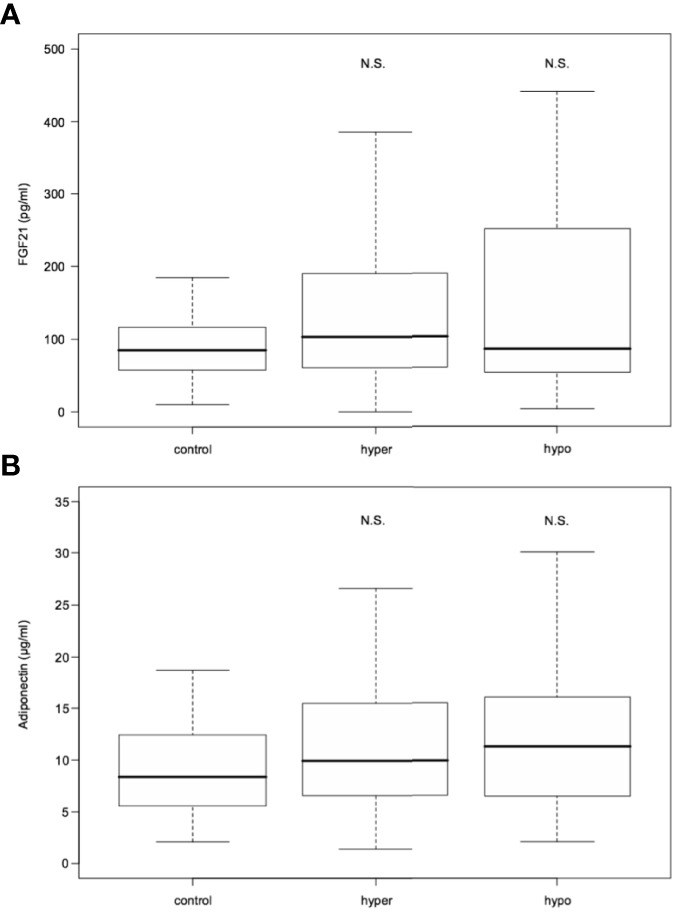
FGF21 **(A)** and adiponectin **(B)** serum levels in hyperthyroid and hypothyroid patients in comparison to controls. FGF21, fibroblast growth factor 21. Data presented as median (interquartile range). N.S. represents non-statistically significant difference. P<0.05 was considered significant.

Serum FGF21 levels did not differ significantly in hyperthyroid and hypothyroid patients in comparison to healthy subjects [median 103.25 (interquartile range, 60.90- 189.48) and 86.10 (54.05-251.02) vs 85.20 (58.00-116.80) pg/mL; *P*=0.200 and 0.503, respectively] (**Table 1** and **Figure 2**). Similarly, serum adiponectin concentrations in hyper- and hypothyroidism did not differ as compared to controls [mean 11.67 ± 7.26 and 13.09 ± 8.34 vs 9.75 ± 5.57 µg/mL; *P*=0.171 and 0.195, respectively] (**Table 1** and **Figure 2**).

### Serum FGF21 Level Rose in Rapid-Onset Hypothyroidism After RAI Treatment

**Figure 3:**
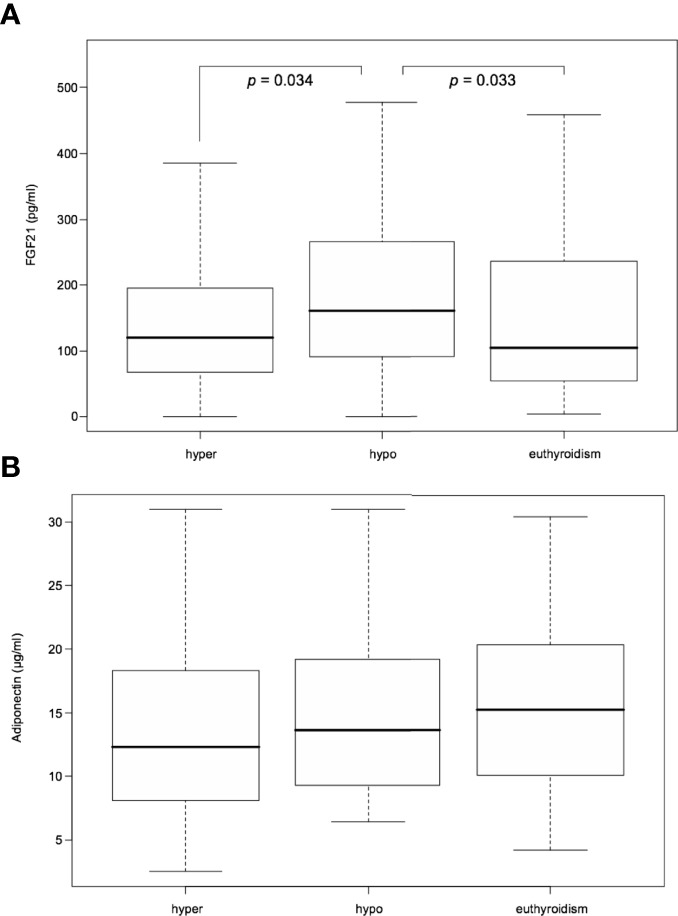
Serum FGF21 **(A)** and adiponectin **(B)** levels after RAI treatment in hyper-, hypo-, and euthyroid phase. FGF21, fibroblast growth factor 21. Data presented as median (interquartile range). P<0.05 was considered significant.

From among the RAI treated hyperthyroid patients, hypothyroidism occurred within one year following therapy in 42 cases. In these patients serum FGF21 levels were unchanged in hyperthyroidism as compared with euthyroidism. However, there was a significant transient increase in FGF21 serum levels in rapid-onset hypothyroidism in comparison to hyperthyroid and euthyroid phase [median 160.55 (interquartile range, 92.48-259.35) vs 119.55 (67.78-192.32) and 104.43 (55.93-231.93) pg/mL, *P*=0.034 and 0.033, respectively] (**Table 2** and **Figure 3**). Furthermore, adiponectin serum concentration in rapid-onset hypothyroidism, hyperthyroidism and euthyroidism were comparable [mean 15.16 ± 6.75 vs 13.62 ± 7.16 and 16.11 ± 6.87 µg/mL, *P=* 0.076 and 0.913, respectively] (**Table 2** and **Figure 3**). Moreover, no significant differences in serum levels of FGF21 or adiponectin occurred between the hyper- and euthyroid phases in the thionamide treated group (**Suppl. Table 5**).

**Table 2:** Serum FGF21 and biochemical parameters after RAI treatment in hyper-, hypo-, and euthyroid phase.

	Hyper-thyroidism	Hypo-thyroidism	Eu-thyroidism	*P^α^*	Hypo vs. Hyper		Eu vs. Hyper		Eu vs. Hypo	
					*MD* (95% *CI*)	*P*	*MD* (95% *CI*)	*P*	*MD* (95% *CI*)	*P*
*N*	42	42	42							
FGF21, pg/mL	119.55 (67.78-192.32)	160.55 (92.48-259.35)	104.43 (55.93-231.93)	-	41.00 (4.75-112.60)	0.034	-15.12 (-65.85-33.80)	>0.999	-56.12 (-83.30-2.90)	0.033
Adiponectin, µg/mL	13.62 ± 7.16	15.16 ± 6.75	16.11 ± 6.87	0.033	1.55 (-0.22-3.18)	0.076	2.50 (-0.47-4.53)	0.051	0.95 (-0.96-2.99)	0.913
TSH, mIU/L	0.00 (0.00-0.00)	32.05 (18.71-49.37)	1.10 (0.43-4.48)	-	32.04 (25.80-41.20)	<0.001	1.10 (0.15-4.95)	<0.001	-30.95 (-38.40-21.85)	<0.001
fT4, pmol/L	57.11 ± 28.66	5.17 ± 2.73	16.07 ± 4.01	<0.001	-51.94 (-62.33-45.12)	<0.001	-41.04 (-51.91-33.86)	<0.001	10.90 (9.09-12.22)	<0.001
fT3, pmol/L	20.86 ± 10.29	2.02 ± 1.10	4.0 ± 0.53	<0.001	-18.83 (-21.1-15.28)	<0.001	-16.76 (-19.86-13.62)	<0.001	2.07 (1.67-2.36)	<0.001
SHBG, nmol/L	160.65 (109.53-187.55)	38.60 (28.73-52.18)	66.40 (50.40-99.00)	-	-122.0 (-141.20-88.60)	<0.001	-94.25 (-135.2-49.90)	<0.001	27.80 (21.60-35.90)	<0.001
Fasting serum insulin,mU/L	7.73 ± 4.83	7.06 ± 3.60	6.66 ± 4.04	0.242						
Fasting plasma glucose, nmol/L	5.25 ± 0.4	4.88 ± 0.6	5.11 ± 0.51	<0.001	-0.37 (-0.59-0.21)	<0.001	-0.14 (-0.3-0.03)	0.051	0.23 (0.08-0.39)	0.014
Quicki	0.37 ± 0.04	0.37 ± 0.04	0.37 ± 0.04	0.456						
HOMA-IR	1.75 ± 1.10	1.55 ± 0.82	1.55 ± 1.03	0.518						
TC, mmol/L	3.98 ± 0.95	7.19 ± 1.59	5.29 ± 1.09	<0.001	3.19 (2.84-3.49)	<0.001	1.29 (1.02-1.68)	<0.001	-1.90 (-2.21-1.49)	<0.001
HDL-c, mmol/L	1.48 ± 0.47	2.26 ± 0.56	1.79 ± 0.46	<0.001	0.78 (0.68-0.96)	<0.001	0.30 (0.21-0.47)	<0.001	-0.47 (-0.59-0.36)	<0.001
LDL-c, mmol/L	1.96 ± 0.72	4.05 ± 1.26	2.86 ± 0.94	<0.001	2.08 (1.71-2.3)	<0.001	0.89 (0.62-1.23)	<0.001	-1.19 (-1.46-0.77)	<0.001
TG, mmol/L	1.04 (0.84-1.34)	1.87 (1.32-2.55)	1.16 (0.85-1.45)	-	0.82 (0.47-0.89)	<0.001	0.11 (-0.05-0.22)	0.663	-0.71 (-0.80-0.37)	<0.001
BMI, kg/m^2^	22.76 ± 3.89	24.48 ± 3.69	24.50 ± 4.40	<0.001	1.73 (1.26-2.28)	<0.001	1.75 (1.02-2.43)	<0.001	0.02 (-0.38-0.44)	>0.999

FGF21, fibroblast growth factor 21; TSH, thyroid stimulating hormone; fT4, free thyroxine; fT3, free triiodothyronine; SHBG, sex hormone binding globulin; Quicki, quantitative insulin-sensitivity check index; HOMA-IR, homeostasis model assessment of insulin resistance; TC, total cholesterol; HDL-c, high density lipoprotein cholesterol; LDL-c, low density lipoprotein cholesterol; TG, triglycerides; BMI, body mass index. Data shown are mean ± SD and median (interquartile range). MD values are mean/median differences between characteristics in different thyroid function phases with 95% confidence interval (CI).

Pα-values are based on results of repeated measures ANOVA.

P-values are based on comparison of characteristics between thyroid function phases with post-hoc test for ANOVA or Wilcoxon paired test.

### Elevated Serum FGF21 in Rapid-Onset Hypothyroidism After RAI Treatment Correlated Positively With Triglycerides, and Negatively With SHBG

**Figure 4:**
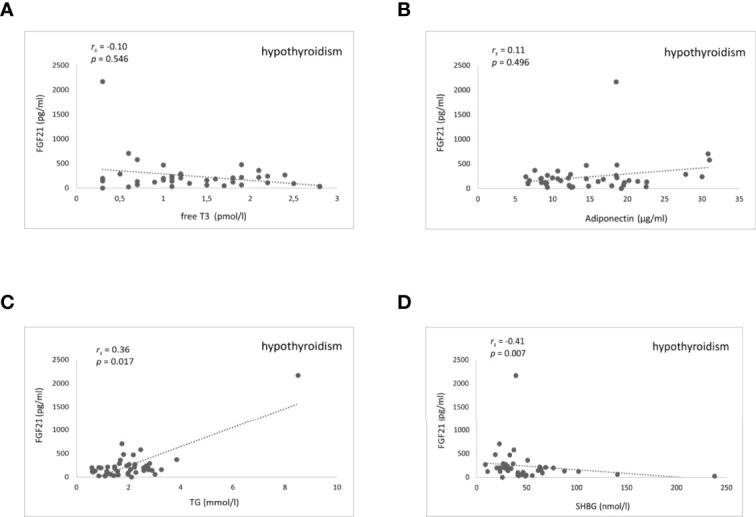
Scatter plots showing FGF21 and fT3, adiponectin, triglycerides, and SHBG serum levels in hypothyroidism after RAI treatment. Relationship between serum FGF21 levels and fT3 **(A)** adiponectin **(B)** triglycerides **(C)** and SHBG **(D)**. FGF21, fibroblast growth factor 21; fT3, free triiodothyronine; TG, triglycerides; SHBG, sex hormone binding globulin. r_s_ represents Spearman correlation coefficient. P<0.05 was considered significant.

There was no significant correlation between FGF21 serum levels and TH in any of the thyroid function states. Furthermore, there were no significant correlations found between serum FGF21 levels and lipids, as well as glucose metabolism parameters in patients with hyperthyroidism (**Suppl. Table 8**). On the contrary, during rapid-onset hypothyroidism following RAI treatment, rising serum FGF21 levels correlated significantly and positively with rising serum triglycerides (Spearman coefficient rs=0.36, *P*=0.017) and inversely with falling serum SHBG (rs=-0.41, *P*=0.007) (**Figure 4** and **Suppl. Table 9**). The correlation disappeared during the subsequent euthyroid phase (**Suppl. Table 10**). Additionally, there were no relationships found of serum FGF21 levels and cholesterol, as well as glucose metabolism parameters during RAI-induced hypothyroidism (**Suppl. Table 9**). Furthermore, serum FGF21 levels did not show any significant correlation with the estimated variables in the euthyroid phase (**Suppl. Table 10**). Moreover, there were no significant correlations observed between serum FGF21 concentration and body composition parameters in women and men in any of the thyroid function states (**Suppl. Tables 11**–**16**).

### There Was no Significant Correlation Between Serum FGF21 and Adiponectin

We did not observe correlations between FGF21 and adiponectin in any of the thyroid function states. Unlike FGF21, there was no correlation found of serum adiponectin, and TG, as well as SHBG concentrations in rapid-onset hypothyroidism after RAI treatment. In hyper- and hypothyroidism, adiponectin correlated positively with age (rs=0.26, *P*=0.020 and rs=0.43, *P*=0.004, respectively), although this relationship disappeared in the euthyroid phase. There was significant positive correlation with HDL-c in hyper-, hypo- and euthyroidism (rs=0.49, *P*<0.001, rs=0.44, *P*=0.003, rs=0.49, *P*=0.001, respectively), with total cholesterol in hyperthyroidism (rs=0.32, *P*=0.004) and with total cholesterol and LDL-c in hypothyroidism (rs=0.48, *P*=0.001 and rs=0.35, *P*=0.020, respectively). Moreover, adiponectin correlated inversely with BMI in the euthyroid phase (rs=-0.33, *P*=0.039) which was not seen in hyper- and hypothyroidism (**Suppl Tables 8**–**10**). In hyper and hypothyroid females, adiponectin correlated negatively with BMC (rs=-0.30, P=0.012 and rs=-0.49, P=0.002, respectively), whereas in the euthyroid phase the inverse correlation between adiponectin and both BMC (rs=-0.46, *P*=0.007) and LTM (rs=-0.37, *P*=0.036) occurred (**Suppl Tables 11**–**16**).

## Discussion

To the best of our knowledge, this study is the first to examine TH influence on FGF21 secretion in a pairwise comparison of three different thyroid functions, namely hyper-, hypo-, and euthyroidism. In our group of RAI-treated patients, dramatic thyroid and metabolic changes occurred in a short period of time, which constitutes a favorable model of FGF21 regulatory pathways. Moreover, because of the wide interindividual variation in FGF21 serum levels in the general population, pairwise comparison represents a more reliable method of FGF21 assessment than intercohort comparison (40). However, even in these favorable settings, we did not demonstrate a stimulatory effect of TH on FGF21 release. Furthermore, we did not observe any correlation between FGF21 and TH in hyper-, hypo-, and euthyroidism.

Our results stay in agreement with studies by Bonde and colleagues, in which neither reduction in FGF21 serum level in 20 hyperthyroid patients after achieving euthyroidism was seen, nor treatment of 14 healthy subjects with liver-specific beta thyroid receptor analog eprotirome cause any change in FGF21 serum level (6). On the contrary, Xiao and colleagues demonstrated elevated FGF21 serum levels in 119 Graves’ disease hyperthyroid patients compared to healthy controls. In a subset of 41 patients from among this group, circulating FGF21 concentration declined by 59% after three months of thionamide treatment when euthyroidism had been established (7). In both studies, there was no correlation between FGF21 and T3 in hyperthyroidism, which suggests no cause-and-effect relationship and is consistent with our results.

Interestingly, both animal and cell culture studies demonstrate interrelation between TH and FGF21 secretion. It has been confirmed that T3 induces transcription of the *Fgf21* gene in mouse liver and human HepG2-TRβ hepatocyte culture (4). This effect is mediated through thyroid hormone receptor-β activation (TRβ) (5). However, expression of most hepatic T3-regulated genes is induced independently of FGF21 and ensues equally in both wild-type and a mouse strain with targeted deletion of *Fgf21* gene (5). This leads to the conclusion that FGF21 is dispensable for the majority of TH action in the liver.

Although we did not find a relationship between hyperthyroidism and FGF21 secretion, we have observed a transient FGF21 rise during rapid-onset hypothyroidism after RAI treatment. In this setting, FGF21 serum level positively correlated with rising serum triglycerides, and negatively with SHBG, which was typically reduced in hypothyroidism. Additionally, no correlation was found between serum FGF21 levels and TH. Because FGF21 strongly induces hepatic FFA oxidation (19), we hypothesized that sudden β-oxidation breakdown due to TH decline triggers FGF21 release as a compensatory reaction.

TH are a key player in FFA metabolism. The mainstay of this action is the induction of carnitine palmitoyltransferase- Iα (CPT-Iα), which is the rate-limiting enzyme in the hepatic β-oxidation pathway (41, 42). CPT-Iα converts long-chain fatty acyl-CoAs to acylcarnitines for transport across the mitochondrial membrane for β-oxidation (43). We hypothesized, that in rapid-onset hypothyroidism, CPT-Iα activity suddenly declines with a subsequent inhibition of FFA oxidation and increased hepatic lipogenesis. In this setting, the rise in serum triglycerides is a marker of intrahepatic mitochondrial β-oxidation breakdown and induction of lipogenesis. Moreover, sudden SHBG reduction in the hypothyroid state may favor hepatic lipid accretion. It has been demonstrated that enhanced *de novo* lipogenesis in the liver, a process consisting of conversion of acetyl-coenzyme A to free fatty acids, is crucial for NAFLD development and can down regulate SHBG synthesis (44, 45). Additionally, in human liver biopsy samples, enhanced TG accumulation and acetyl-coenzyme A carboxylase (ACC) mRNA expression, being the rate limiting enzyme controlling *de novo* lipogenesis, correlates negatively with SHBG mRNA and protein levels. Furthermore, SHBG overexpression decreases ACC expression induced by high-glucose culture conditions in HepG2 cells (46).. This implicates a protective function of SHBG in a fatty liver formation and is consistent with our results, where falling SHBG serum concentration associated with FGF21 release.

It had been shown previously that exogenous administration of FGF21 significantly lowers circulating and intrahepatic triglycerides, reverses fatty liver, and reduces signs of NASH and hepatic fibrosis (23). Administration of the FGF21 analogue pegbelfermin in humans with NASH causes at least a 30% relative reduction in hepatic fat content assessed in magnetic resonance imaging-proton density fat fraction in over half of the patients and a significant reduction of serum triglyceride levels (23). FGF21 increases fatty acid oxidation in the liver but also reduces lipid flux into the liver by promoting triglyceride-rich lipoproteins catabolism in adipose tissue and suppression of white adipose tissue postprandial lipolysis (19–21). However, these data emerge from animal experiments or human studies after exogenous administration of FGF21 analogues, which do not fully reflect physiology. In our study, we confirmed the physiological endogenous rise of FGF21 secretion accompanying sudden lipid overload, demonstrating its protective function against mitochondrial stress. Indeed, hepatic transcription of *fgf21* gene in mice is triggered by endoplasmic reticulum (17) and oxidative stress (16) resulting from obesity and NAFLD as a compensatory reaction. On the other hand, FGF21 resistance due to attenuated receptor signaling and consequently impaired induction of target genes in diet-induced obesity (DIO) mice has also been described (38). It leads to the conclusion that both mechanisms can be involved and increased FGF21 serum levels in obesity related comorbidities may be a concert of compensatory increased secretion and receptor resistance.

In our study, we did not observe the link between FGF21 and adiponectin. In rapid-onset hypothyroidism after RAI treatment, rising FGF21 serum concentration was not accompanied by augmented adiponectin secretion. Moreover, adiponectin did not correlate with rising FGF21 serum levels. Therefore, we have not confirmed the hypothesis that the hepatic action of FGF21 is mediated indirectly *via* increased adiponectin secretion (27), a result that is consistent with other studies demonstrating adiponectin is dispensable for FGF21 action (28).

One limitation of our study was the relatively small sample size and inequality of sex distribution, reducing the strength of the sex-specific body composition assessment.

In conclusion, this study did not exhibit interdependency between TH and FGF21 secretion. Serum FGF21 concentrations in hyper- and hypothyroidism did not differ in comparison to the control group. Furthermore no correlation between FGF21 and T3 has been demonstrated.

Instead, we observed a significant rise of FGF21 serum level in conditions of metabolic challenge of rapid-onset hypothyroidism after RAI treatment. This rise demonstrated a significant positive correlation with rising serum triglyceride and negative correlation with falling SHBG concentration. We conclude that FGF21 induction in physiological conditions of increased hepatic lipogenesis suggests a compensatory reaction protecting the liver against excessive triglyceride accumulation.

## Data Availability Statement

The datasets presented in this study can be found in online repositories. The names of the repository/repositories and accession number(s) can be found in the article/**Supplementary Material**.

## Ethics Statement

The studies involving human participants were reviewed and approved by Bioethical Committee of Centre of Postgraduate Medical Education, Warsaw, Poland. The patients/participants provided their written informed consent to participate in this study.

## Author Contributions

ES, WZ and MG-C designed the project. The first draft of the manuscript was written by ES. ES, WZ, JS-S, HJ, and MG-C wrote, reviewed, and edited the manuscript. Data collection was performed by ES. PG performed laboratory measurements and reviewed the manuscript. ES performed the statistical analysis. All authors contributed to the final version of the manuscript and approved it for publication.

## Funding

This study was supported by grant of the Centre of Postgraduate Medical Education (No. 506-1-08-02).

## Conflict of Interest

The authors declare that the research was conducted in the absence of any commercial or financial relationships that could be construed as a potential conflict of interest.

## Publisher’s Note

All claims expressed in this article are solely those of the authors and do not necessarily represent those of their affiliated organizations, or those of the publisher, the editors and the reviewers. Any product that may be evaluated in this article, or claim that may be made by its manufacturer, is not guaranteed or endorsed by the publisher.
