# Supplementary material for: FGF21 Is Released During Increased Lipogenesis State Following Rapid-Onset Radioiodine-Induced Hypothyroidism
Source: Front Endocrinol (Lausanne). 2022 Jul 14;13:900034. doi: 10.3389/fendo.2022.900034 (PMC9329662; doi:10.3389/fendo.2022.900034)
Supplement: Supplementary file 1 [file DataSheet_1.pdf]

	controls	hyperthyroidism	P^{α}	hypothyroidism	P^{β}
n	21	72		11	
Legs BF, %	35.81±8.37	38.58±7.37	0.212	32.03±9.94	0.306
Trunk BF,%	35.66±12.69	37.88±8.90	0.492	35.75±12.43	0.985
BF, %	35.81±10.02	37.80±7.67	0.440	33.94±10.75	0.646
Legs BF, kg	7.74±3.28	7.78±2.58	0.963	6.85±3.51	0.510
Trunk BF, kg	11.32±6.17	11.10±4.27	0.892	11.50±6.24	0.939
BF, kg	23.83±11.16	23.27±8.05	0.842	22.47±11.17	0.755
LTM, kg	39.83±4.38	36.69±4.77	0.013	40.07±4.46	0.891
BMC, kg	2.52±0.53	2.45±0.46	0.586	2.43±0.45	0.661
Waist cirumference,cm	81.90±12.03	79.09±9.88	0.380	86.09±14.78	0.432

Table 1. Body composition parameters in hyperthyroid and hypothyroid women and the control group women .
Legs BF, legs body fat; Trunk BF, trunk body fat; BF, body fat; LTM, lean tissue mass; BMC, bone mineral content. Data shown are mean ± SD.
Pα-values are based on comparison of characteristics between hyperthyroid females vs controls.
Pβ-values are based on comparison of characteristics between hypothyroid females vs controls.

	controls	hyperthyroidism	P^{α}	hypothyroidism	P^{β}
n	4	10		4	
Legs BF,%	16.90 (16.28-7.83)	24.55 (20.68-27.58)	0.106	22.35 (18.38-25.98)	0.343
Trunk BF,%	27.20 (24.05-29.25)	27.55 (25.25-33.60)	0.671	31.05 (26.60-36.60)	0.486
BF,%	21.25 (19.85-22.70)	25.55 (22.15-29.63)	0.304	26.05 (21.45-31.75)	0.384
Legs BF, kg	3.97 (3.57-4.40)	5.53 (4.74-7.15)	0.119	4.66 (3.41-6.28)	>0.999
Trunk BF, kg	9.30 (8.14-10.12)	9.62 (8.92-13.31)	0.571	11.31 (8.42-15.26)	0.486
BF, kg	16.53 (14.97-17.00)	17.99 (15.96-23.67)	0.188	20.11 (14.13-27.14)	0.886
LTM, kg	56.17 (52.50-60.48)	56.48 (53.37-57.86)	0.839	56.06 (51.51-58.07)	0.686

BMC, kg	2.91 (2.89-3.08)	3.13 (2.94-3.29)	0.390	2.96 (2.64-3.11)	>0.999
Waist cirumference,cm	90.00 (89.50-90.50)	91.00 (86.25-97.25)	>0.999	90.00 (86.50-93.00)	>0.999

Table 2. Body composition parameters in hyperthyroid and hypothyroid men and the control group men.
Legs BF, legs body fat; Trunk BF, trunk body fat; BF, body fat; LTM, lean tissue mass; BMC, bone mineral content. Data shown are median (interquartile range).
Pα-values are based on comparison of characteristics between hyperthyroid males vs controls.
Pβ-values are based on comparison of characteristics between hypothyroid males vs controls.

	**Hyper thyroidism**	**Hypo thyroidism**	**Eu thyroidism**	$\boldsymbol{P}^{\alpha}$	**Hypo vs. Hyper**		**Eu vs. Hyper**		**Eu vs. Hypo**	
					MD (95% *CI*)	*P*	*MD* (95% *CI*)	*P*	*MD* (95% *CI*)	*P*
n	38	38	38							
Legs BF,%	38.85±7.97	37.78±8.31	38.05±8.76	0.341						
Trunk BF,%	38.41±9.53	38.05±8.30	38.36±9.28	0.904						
BF,%	38.08±8.30	37.53±7.54	37.85±8.42	0.992						
Legs BF, kg	7.60±2.76	7.90±2.99	8.13±3.30	0.013	0.30 (-0.14-0.67)	0.582	0.53 (-0.03-0.97)	0.110	0.23 (-0.20-0.30)	>0.999
Trunk BF, kg	10.97±4.46	11.72±4.29	11.83±4.75	0.008	0.75 (0.26-1.57)	0.022	0.86 (-0.08-1.49)	0.085	0.12 (-0.88-0.43)	>0.999
BF, kg	22.84±8.56	23.95±8.24	24.58±9.43	0.006	1.11 (-0.09-2.35)	0.103	1.73 (0.33-2.91)	0.046	0.62 (-0.86-0.93)	>0.999
LTM, kg	35.26±4.62	38.39±4.97	38.33±4.99	<0.001	3.13 (2.59-3.87)	<0.001	3.07 (1.96-3.35)	<0.001	-0.06 (-1.21–0.04)	0.043
BMC, kg	2.36±0.50	2.27±0.47	2.35±0.52	0.009	-0.08 (-0.11--0.03)	0.001	-0.01 (-0.06-0.01)	0.521	0.08 (-0.01-0.16)	0.440

	Hyper thyroidism	Hypo thyroidism	Eu thyroidism	P^{α}	Hypo vs. Hyper		Eu vs. Hyper		Eu vs. Hypo	
					MD (95% *CI*)	*P*	*MD* (95% *CI*)	*P*	*MD* (95% *CI*)	*P*
Waist ci-rumference, cm	79.11±10.27	82.88±10.07	81.56±11.46	0.314						

Table 3. Body composition parameters in women treated with radioiodine in hyper-, hypo-, and euthyroid phase.

Legs BF, legs body fat; Trunk BF, trunk body fat; BF, body fat; LTM, lean tissue mass; BMC, bone mineral content. Data shown are mean ± SD. MD values are mean differences between characteristics in different thyroid function phases with 95% confidence interval (CI).

Pα-values are based on results of ANOVA for repeated measures.

P-values are based on comparison of characteristics between thyroid function phases with post-hoc test for ANOVA .

	Hyper thyroidism	Hypo thyroidism	Eu thyroidism	Hypo vs. Hyper		Eu vs. Hyper		Eu vs. Hypo	
				MD (95% *CI*)	*P*	*MD* (95% *CI*)	*P*	*MD* (95% *CI*)	*P*
n	4	4	4						
Legs BF,%	28.00 (25.33-30.68)	28.10 (27.95-31.25)	28.50 (25.05-32.08)	0.10 (-2.60-1.20)	>0.999	0.50 (-0.10-1.40)	>0.999	0.40 (-1.60-2.70)	>0.999
Trunk BF,%	28.50 (27.13-31.78)	38.00 (33.95-39.20)	38.20 (35.33-39.68)	9.50 (-4.70-12.30)	>0.999	9.70 (1.30-13.80)	0.375	0.20 (-0.20-7.00)	>0.999
BF,%	28.20 (26.50-29.98)	34.80 (31.05-35.30)	33.30 (30.55-34.93)	6.60 (-4.30-7.00)	>0.999	5.10 (0.70-8.30)	0.375	-1.50 (-5.60-5.10)	>0.999
Legs BF, kg	7.40 (6.81-7.61)	7.64 (6.54-8.44)	7.91 (7.07-8.79)	0.24 (-0.50-1.00)	0.815	0.52 (0.10-2.10)	0.375	0.28 (-0.90-1.60)	>0.999
Trunk BF, kg	9.78 (8.92-11.26)	12.97 (11.68-14.98)	14.29 (13.26-15.98)	3.20 (-1.60-6.50)	0.563	4.52 (-1.90-7.70)	0.375	1.32 (-0.20-3.70)	0.750
BF, kg	19.11 (18.20-22.03)	24.37 (20.44-29.38)	28.43 (24.97-30.85)	5.26 (-1.60-9.80)	0.563	9.32 (3.00-12.00)	0.375	4.06 (-0.80-6.50)	>0.999

	Hyper thyroidism	Hypo thyroidism	Eu thyroidism	Hypo vs. Hyper		Eu vs. Hyper		Eu vs. Hypo	
				MD (95% *CI*)	*P*	*MD* (95% *CI*)	*P*	*MD* (95% *CI*)	*P*
LTM, kg	54.58 (50.32-57.4)	57.65 (52.73-61.98)	58.33 (54.23-61.71)	3.08 (-1.60-7.70)	0.188	3.76 (-3.20-6.10)	0.375	0.68 (-0.80-1.60)	>0.999
BMC, kg	2.90 (2.86-3.02)	2.90 (2.87-3.08)	3.12 (3.06-3.22)	0.00 (-0.10-0.20)	>0.999	0.22 (-0.20-0.60)	0.792	0.22 (-0.20-0.60)	>0.999
Waist ci-rumference, cm	94.00 (87.50-105.0)	96.00 (92.00-102.50)	101.00 (97.00-105.00)	2.00 (-2.00-2.00)	0.521	7.00 (-2.00-12.00)	0.750	5.00 (-2.00-8.00)	>0.999

Table 4. Body composition parameters in men treated with radioiodine in hyper-, hypo-, and euthyroid phase.
Legs BF, legs body fat; Trunk BF, trunk body fat; BF, body fat; LTM, lean tissue mass; BMC, bone mineral content.
Data shown are median (interquartile range). MD values are median differences between characteristics in different thyroid function phases with 95% confidence interval (CI).
P-values are based on comparison of characteristics between thyroid function phases with Wilcoxon paired test.

	Hyperthyroidism	Euthyroidism	***MD* (95% *CI*)**	***P***
n	17	17		
TSH, mIU/L	0.00 (0.00-0.00)	0.71 (0.10-2.32)	0.71 (0.69-2.75)	0.001
fT4,pmol/L	51.81±31.35	12.18±2.05	-39.62 (-55.67- –23.59)	<0.001
fT3, pmol/L	20.35±11.85	4.22±0.79	-16.12 (-22.21- –10.04)	<0.001
SHBG, nmol/L	174.95 (140.50-240.00)	70.35 (45.73-88.25)	-104.60 (-161.85- -61.80)	<0.001
Fasting serum insulin, mU/L	5.80±1.85	6.61±3.51	0.80 (-0.88-2.48)	0.329
Fasting plasma glucose, nmol/L	5.40±0.64	5.17±0.61	-0.22 (-0.60-0.15)	0.232
Quicki	0.37±0.02	0.37±0.03	0.00 (-0.06-0.01)	0.055
HOMA-IR	1.39±0.46	1.49±0.86	0.10 (-0.31-0.53)	0.575

TC,mmol/L	3.5±0.82	4.91±0.94	1.41 (0.82-2.00)	<0.001
HDL-c, mmol/L	1.20±0.26	1.61±0.31	0.41 (0.27-0.55)	<0.001
LDL-c, mmol/L	1.77±0.62	2.79±0.85	1.02 (0.55-1.49)	<0.001
TG, mmol/L	1.14±0.45	1.08±0.42	-0.06 (-0.33-0.21)	0.635
BMI, kg/m^2	23.09±3.76	24.69±4.03	1.60 (1.06-2.16)	<0.001
Adiponectin,**μ**gmL	3.18±0.72	7.76±3.06	4.58 (-1.22-10.42)	0.077
FGF21,pg/mL	68.40 (44.10-140.90)	95.60 (62.80-122.50)	27.20 (-17.75-43.25)	0.677

Table 5. Serum FGF21, clinical and biochemical characteristics in patients treated with thionamides pre-treatment and three months after euthyroidism has been established .

TSH, thyroid stimulating hormone; fT4, free thyroxine, fT3, free triiodothyronine; SHBG, sex hormone binding globulin; QUICKI, quantitative insulin-sensitivity check index; HOMA-IR, homeostasis model assessment of insulin resistance; TC, total cholesterol; HDL-c, high density lipoprotein cholesterol; LDL-c, low density lipoprotein cholesterol; TG, triglycerides, BMI, body mass index; FGF21, fibroblast growth factor 21. Data shown are mean ± SD or median (interquartile range).
MD values are mean/median differences between characteristics in different thyroid function phases with 95% confidence interval (CI).
P-values are based on comparison of characteristics between thyroid function phases with paired T-test or Wilcoxon paired test.

	**Hyperthyroidism**	**Euthyroidism**	***MD*** **(95%** ***CI*****)**	*P*
n	14	14		
Legs BF, %	38.67±6.08	39.01±6.57	0.34 (-1.26-1.93)	0.649
Trunk BF, %	36.35±10.01	37.19±9.38	0.84 (-0.82-2.49)	0.287
BF, %	36.93±8.18	37.87±7.57	0.95 (-0.58-2.31)	0.213
Legs BF, kg	7.84±2.59	8.77±3.14	0.92 (0.35-1.54)	0.005
Trunk BF, kg	10.79±5.11	11.54±4.81	0.75 (-0.07-1.58)	0.068
BF, kg	22.88±9.15	25.26±9.47	2.38 (1.08-3.70)	0.002
LTM, kg	37.12±3.48	39.52±3.58	2.40 (1.20-3.60)	0.001
BMC, kg	2.75±0.34	2.75±0.28	0.00 (-0.08-0.08)	>0.999
Waist cirumference, cm	72.00 (67.50-75.50)	73.00 (71.00-80.50)	1.00 (-3.50-13.00)	0.150

Table 6. Body composition parameters in women treated with thionamides pre-treatment and three months after euthyroidism has been established .

Legs BF, legs body fat; Trunk BF, trunk body fat; BF, body fat; LTM, lean tissue mass; BMC, bone mineral content.

Data shown are mean±SD. MD values are median differences between characteristics in different thyroid function phases with 95% confidence interval (CI).

P-values are based on comparison of characteristics between thyroid function phases with paired T-test or Wilcoxon paired test.

	**Hyperthyroidism**	**Euthyroidism**	***MD* (95% *CI*)**	***P***
n	3	3		
Legs BF, %	12.80 (11.35-19.05)	14.50 (11.75-21.25)	1.70 (-3.45-5.78)	0.391
Trunk BF, %	22.60 (19.25-28.80)	25.70 (19.15-32.10)	3.10 (-8.38-0.58)	0.667
BF, %	17.50 (15.15-23.70)	19.40 (14.80-25.75)	1.90 (-6.18-7.18)	0.778
Legs BF, kg	3.32 (2.66-4.90)	4.53 (3.23-6.37)	1.21 (-1.37-3.24)	0.224
Trunk BF, kg	9.00 (7.04-11.70)	10.90 (7.37-13.14)	1.90 (-3.57-4.63)	0.632
BF, kg	14.27 (11.24-19.62)	17.74 (12.12-22.58)	3.48 (-5.35-8.08)	0.473
LTM, kg	58.53 (57.18-62.99)	57.98 (57.49-65.83)	-0.55 (-10.35-6.95)	0.371
BMC, kg	3.22 (3.17-3.58)	3.29 (3.22-3.67)	0.07 (-0.01-0.09)	0.174

Table 7. Body composition parameters in men treated with thionamides pre-treatment and three months after euthyroidism has been established .

Legs BF, legs body fat; Trunk BF, trunk body fat; BF, body fat; LTM, lean tissue mass; BMC, bone mineral content.

Data shown are median (interquartile range). MD values are median differences between characteristics in different thyroid function phases with 95% confidence interval (CI).

P-values are based on comparison of characteristics between thyroid function phases with Wilcoxon paired test.

	**FGF21**		**Adiponectin**	
	r_s	*P*	r_s	*P*
n	82		80	
FGF21	-	-	-0.03	0.823
Adiponectin	-0.03	0.823	-	-
TSH	-0.05	0.660	-0.10	0.402
fT4	0.08	0.486	-0.03	0.788
fT3	-0.05	0.669	-0.13	0.255

	FGF21		Adiponectin	
	r_s	*P*	r_s	*P*
SHBG	0.12	0.296	-0.16	0.164
Fasting serum insulin	-0.15	0.178	0.04	0.759
Fasting plasma glucose	0.03	0.785	-0.01	0.914
Quicki	0.15	0.177	-0.01	0.900
HOMA-IR	-0.13	0.248	0.01	0.996
TC	-0.08	0.492	0.32	0.004**
HDL-c	-0.06	0.607	0.49	<0.001***
LDL-c	-0.07	0.553	0.17	0.132
TG	0.08	0.489	-0.09	0.426
BMI	0.03	0.825	-0.08	0.468
Age	0.11	0.313	0.26	0.020*

Table 8 Correlations of FGF21 and adiponectin with clinical and biochemical characteristics in hyperthyroidism.
TSH, thyroid stimulating hormone; fT4, free thyroxine, fT3, free triiodothyronine; SHBG, sex hormone binding globulin; QUICKI, quantitative insulin-sensitivity check index; HOMA-IR, homeostasis model assessment of insulin resistance; TC, total cholesterol; HDL-c, high density lipoprotein cholesterol; LDL-c, low density lipoprotein cholesterol; TG, triglycerides, BMI, body mass index; FGF21, fibroblast growth factor 21. r_s *is Spearman's correlation coefficient*
*P-values are based on correlation of FGF21 and adiponectin with other parameters. *P<0.05 was considered significant.*

	FGF21		Adiponectin	
	**r_s**	***P***	**r_s**	***P***
	44		44	
FGF21	-	-	0.11	0.496
Adiponectin	0.11	0.496	-	-
TSH	0.14	0.369	-0.02	0.886
fT4	-0.16	0.306	-0.08	0.606
fT3	-0.10	0.546	-0.28	0.074
SHBG	-0.41	0.007**	-0.16	0.326
Fasting serum insulin	0.07	0.666	-0.13	0.397
Fasting plasma glucose	-0.14	0.352	-0.13	0.387
Quicki	-0.22	0.166	0.06	0.689
HOMA-IR	0.07	0.660	-0.15	0.351

TC	0.28	0.065	0.48	0.001**
HDL-c	0.20	0.183	0.44	0.003**
LDL-c	0.16	0.295	0.35	0.020*
TG	0.36	0.017*	0.07	0.635
BMI	0.03	0.864	0.01	0.921
Age	0.23	0.132	0.43	0.004**

Table 9. Correlations of FGF21 and adiponectin with clinical and biochemical characteristics in the hypothyroid phase after radioiodine treatment.

TSH, thyroid stimulating hormone; fT4, free thyroxine, fT3, free triiodothyronine; SHBG, sex hormone binding globulin; QUICKI, quantitative insulin-sensitivity check index; HOMA-IR, homeostasis model assessment of insulin resistance; TC, total cholesterol; HDL-c, high density lipoprotein cholesterol; LDL-c, low density lipoprotein cholesterol; TG, triglycerides, BMI, body mass index; FGF21, fibroblast growth factor 21. r_s is Spearman's correlation coefficient
*P-values are based on correlation of FGF21 and adiponectin with other parameters. *P<0.05 was considered significant.*

	**FGF21**		**Adiponectin**	
	r_s	***P***	r_s	***P***
	43		43	
FGF21	-	-	0.18	0.257
Adiponectin	0.18	0.257	-	-
TSH	-0.06	0.697	0.10	0.527
fT4	0.27	0.083	0.25	0.104
fT3	-0.01	0.930	-0.15	0.347
SHBG	-0.18	0.264	0.18	0.255
Fasting plasma insulin	0.08	0.594	-0.18	0.247
Fasting plasma glucose	0.15	0.340	-0.08	0.625
Quicki	-0.22	0.153	0.06	0.700
HOMA-IR	0.09	0.557	-0.18	0.253
TC	0.12	0.426	0.05	0.732
HDL-c	0.03	0.869	0.49	0.001**
LDL-c	0.05	0.736	-0.20	0.192
TG	0.21	0.182	-0.28	0.073

BMI	-0.12	0.460	-0.33	0.039*
Age	0.15	0.335	0.19	0.231

Table 10. Correlations of FGF21 and adiponectin with clinical and biochemical characteristics in the euthyroid phase after radioiodine treatment.
TSH, thyroid stimulating hormone; fT4, free thyroxine, fT3, free triiodothyronine; SHBG, sex hormone binding globulin; QUICKI, quantitative insulin-sensitivity check index; HOMA-IR, homeostasis model assessment of insulin resistance; TC, total cholesterol; HDL-c, high density lipoprotein cholesterol; LDL-c, low density lipoprotein cholesterol; TG, triglycerides, BMI, body mass index; FGF21, fibroblast growth factor 21. r_s *is Spearman's correlation coefficient*
*P-values are based on correlation of FGF21 and adiponectin with other parameters. *P<0.05 was considered significant.*

	**FGF21**		**Adiponectin**	
	r_s	p	r_s	p
	72		70	
Legs BF, %	0.07	0.576	0.07	0.559
Trunk BF, %	-0.01	0.995	-0.05	0.695
BF, %	0.03	0.795	-0.02	0.904
Legs BF, kg	-0.01	0.982	0.07	0.581
Trunk BF, kg	-0.03	0.805	-0.03	0.805
BF, kg	-0.01	0.928	0.01	0.906
LTM, kg	-0.13	0.296	-0.05	0.688
BMC, kg	-0.19	0.117	-0.30	0.012*
Waist circumference, cm	0.03	0.842	0.09	0.618

Table 11. Correlations of FGF21 and adiponectin with body composition characteristics in hyperthyroid women.
Legs BF, legs body fat; Trunk BF, trunk body fat; BF, body fat; LTM, lean tissue mass; BMC, bone mineral content.
r_s *is Spearman's correlation coefficient.*
*P-values are based on correlation of FGF21 and adiponectin with other parameters. *P<0.05 was considered significant.*

	**FGF21**		**Adiponectin**	
	$\mathbf{r_s}$	$\mathbf{p}$	$\mathbf{r_s}$	$\mathbf{p}$
	10		10	
Legs BF, %	0.43	0.218	0.38	0.279
Trunk BF, %	0.10	0.785	0.09	0.811
BF, %	0.19	0.608	0.04	0.919

Legs BF, kg	0.13	0.713	0.37	0.292
Trunk BF, kg	0.09	0.811	-0.18	0.632
BF, kg	0.22	0.537	0.07	0.865
LTM, kg	-0.50	0.143	-0.41	0.247
BMC, kg	0.10	0.789	-0.68	0.032*
Waist circumference, cm	-0.40	0.750	-0.40	0.750

Table 12. Correlations of FGF21 and adiponectin with body composition characteristics in hyperthyroid men.
Legs BF, legs body fat; Trunk BF, trunk body fat; BF, body fat; LTM, lean tissue mass; BMC, bone mineral content
r_s is Spearman's correlation coefficient.
*P-values are based on correlation of FGF21 and adiponectin with other parameters. *P<0.05 was considered significant.*

	**FGF21**		**Adiponectin**	
	r_s	*P*	r_s	*P*
	39		40	
Legs BF, %	0.06	0.735	0.26	0.113
Trunk BF, %	0.21	0.208	0.14	0.389
BF, %	0.07	0.672	0.18	0.269
Legs BF, kg	-0.05	0.752	0.15	0.364
Trunk BF, kg	0.11	0.496	0.05	0.763
BF, kg	0.01	0.947	0.08	0.636
LTM, kg	-0.15	0.383	-0.21	0.191
BMC, kg	-0.04	0.789	-0.49	0.002**
Waist circumference, cm	0.37	0.065	0.29	0.148

Table 13. Correlations of FGF21 and adiponectin with body composition characteristics in women treated with radioiodine in the hypothyroid phase.
Legs BF, legs body fat; Trunk BF, trunk body fat; BF, body fat; LTM, lean tissue mass; BMC, bone mineral content
r_s is Spearman's correlation coefficient.
*P-values are based on correlation of FGF21 and adiponectin with other parameters.*P<0.05 was considered significant.*

	**FGF21**		**Adiponectin**	
	r_s	***p***	r_s	***p***

	5		4	
Legs BF, %	-0.40	0.750	-0.50	>0.999
Trunk BF, %	-0.40	0.750	-0.99	0.333
BF, %	-0.80	0.333	-0.50	>0.999
Legs BF, kg	0.30	0.683	-0.60	0.417
Trunk BF, kg	0.01	>0.999	-0.99	0.083
BF, kg	-0.10	0.950	-0.99	0.083
LTM, kg	-0.30	0.683	0.01	>0.999
BMC, kg	0.35	0.559	-0.77	0.225
Waist circumference, cm	0.50	>0.999	-0.50	>0.999

Table 14. Correlations of FGF21 and adiponectin with body composition characteristics in men treated with radioiodine in the hypothyroid phase.

Legs BF, legs body fat; Trunk BF, trunk body fat; BF, body fat; LTM, lean tissue mass; BMC, bone mineral content

r_s is Spearman's correlation coefficient.

*P-values are based on correlation of FGF21 and adiponectin with other parameters.*P<0.05 was considered significant*

	**FGF21**		**Adiponectin**	
	r_s	*P*	r_s	*P*
	39		39	
Legs BF, %	-0.02	0.931	-0.16	0.377
Trunk BF, %	0.11	0.535	-0.13	0.454
BF,%	0.02	0.905	-0.13	0.458
Legs BF, kg	-0.06	0.749	-0.27	0.122
Trunk BF, kg	0.11	0.543	-0.18	0.326
BF, kg	-0.02	0.894	-0.23	0.188
LTM, kg	-0.12	0.511	-0.37	0.036*
BMC, kg	-0.21	0.237	-0.46	0.007**
Waist circumference, cm	0.15	0.460	-0.33	0.090

Table 15. Correlations of FGF21 and adiponectin with body composition characteristics in women treated with radioiodine in the euthyroid phase.

Legs BF, legs body fat; Trunk BF, trunk body fat; BF, body fat; LTM, lean tissue mass; BMC, bone mineral content.

*r_s is Spearman's correlation coefficient. P-values are based on correlation of FGF21 and adiponectin with other parameters.*P<0.05 was considered significant.*

	FGF21		Adiponectin	
	r_s	***P***	***r_s***	***P***
	4		4	
Legs BF, %	0.99	0.083	0.20	0.917
Trunk BF, %	0.40	0.750	0.01	>0.999
BF, %	0.80	0.333	0.40	0.750
Legs BF, kg	0.99	0.083	0.20	0.917
Trunk BF, kg	0.40	0.750	0.01	>0.999
BF, kg	0.40	0.750	0.01	>0.999
LTM, kg	-0.80	0.333	-0.40	0.750
BMC, kg	0.32	0.684	-0.32	0.684
Waist circumference, cm	-0.99	0.333	-0.50	>0.999

Table 16. Correlations of FGF21 and adiponectin with body composition characteristics in men treated with radioiodine in euthyroid phase.

Legs BF, legs body fat; Trunk BF, trunk body fat; BF, body fat; LTM, lean tissue mass; BMC, bone mineral content.

r_s is Spearman's correlation coefficient.

*P-values are based on correlation of FGF21 and adiponectin with other parameters.*P<0.05 was considered significant.*
